# Nearly Missed Pharyngeal Foreign Body: A Three-Year-Old van Gogh

**DOI:** 10.7759/cureus.24775

**Published:** 2022-05-06

**Authors:** Philippe Haroun, Paolo Simoni, Anne-Laure Mansbach, Grammatina Boitsios

**Affiliations:** 1 Medical School, Université libre de Bruxelles (ULB), Brussels, BEL; 2 Pediatric Radiology, Hôpital Universitaire des Enfants Reine Fabiola, Brussels, BEL; 3 Otolaryngology, Hôpital Universitaire des Enfants Reine Fabiola, Brussels, BEL

**Keywords:** delayed diagnosis, radiolucent object, pediatric head trauma, head and neck imaging, pharyngeal foreign body

## Abstract

Foreign body injuries in the head and neck region can be life-threatening. Managing pediatric patients in this context may be increasingly challenging due to several medical and legal reasons. In order to optimize the management of foreign body injuries and to guide treatment procedures, various imaging techniques, with specific assets and liabilities, must be employed. Nevertheless, the "As Low As Reasonably Achievable'' principle must be kept in mind when managing pediatric patients since children are more radiosensitive than adults. Guidelines for imaging pediatric head traumas are provided by the American College of Radiology (ACR), relying on the Pediatric Emergency Care Applied Research Network (PECARN) severity classification. We report the case of a three-year-old child in whom a considerable delay occurred in diagnosing a foreign body impaction, due to an occult clinical presentation. In this case study, we focus on outlining the importance of considering advanced imaging investigations for children in the wake of traumatic events.

## Introduction

The pediatric population is notorious for foreign body injuries [1,2,3]. The highest prevalence of foreign body injuries is reported in children between zero and three years [4]. The prognosis varies notably depending on injured structures [2].

The head and neck region is critical since it is a mosaic of vital yet vulnerable structures such as vascular and nervous elements, the aerodigestive tract, and the auditory and visual systems [1]. Foreign body injuries involving this area have potentially life-threatening consequences [1]. Accordingly, medico-legal repercussions must be considered, and thorough investigations must be performed prior to discharge. As a matter of fact, medical malpractice litigation and claims against physicians for complications due to missed foreign bodies have commonly been reported [2].

Apart from acute injuries, long-term complications may arise. For example, persisting pain, restriction of joint movements, impaired wound healing, inflammatory responses with potential abscess formation, fistulas, osteomyelitis, necrotizing fasciitis, and foreign body migration have all been detailed in the literature [5]. Therefore, foreign body injuries in the head and neck area bear the risk of lifelong consequences for the patient.

Early foreign body detection is fundamental in minimizing these complications [4,6]. However, the diagnosis might be tricky in children because of some peculiar aspects, such as restricted patient history and clinical examination, and limited imaging modalities might delay the diagnosis and make it a real challenge [1]. Moreover, symptoms can be nonspecific and thereby misinterpreted by mimicking GI or respiratory infections [4]. On top of that, asymptomatic cases of long-duration impaction have already been described [4,5].

Children's evaluation following head traumas is based upon the American College of Radiology (ACR) appropriateness criteria for head trauma, which rely on the Pediatric Emergency Care Applied Research Network (PECARN) classification [7,8]. Imaging studies are pivotal for assessing head traumas, detecting foreign bodies, determining their subsequent spatial relation to neighboring structures, and planning surgical procedures [1].

The present case illustrates a delayed diagnosis of a foreign body impaction in a three-year-old child who presented to the ER following minor head trauma, with no history of foreign body impaction. At the initial presentation, he had an unremarkable clinical examination and did not meet the PECARN criteria for further imaging evaluation.

## Case presentation

**Figure 1:**
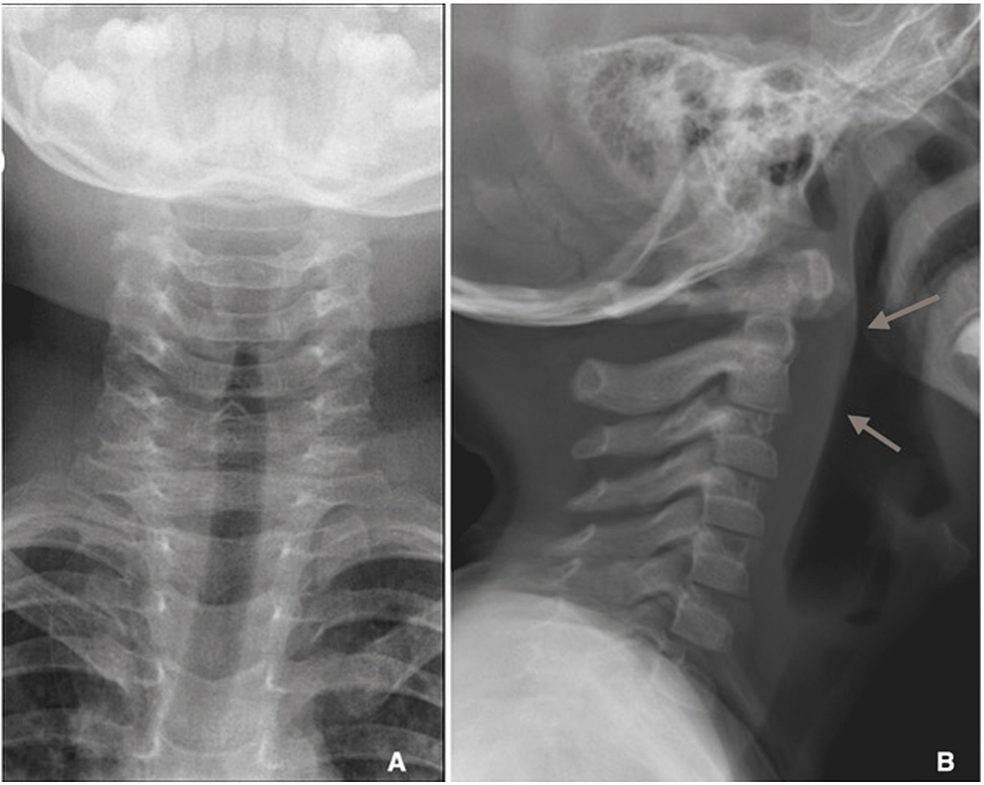
Coronal (A) and lateral (B) standard radiographs of the cervical spine showing non-specific thickening of the prevertebral soft tissues (arrows).

A three-year-old boy presented to the ER for neck pain associated with blood-stained saliva following a head trauma while playing with his older sister. The sister mentioned that he fell off head-first as he sat on her shoulders. Anamnesis revealed there was no loss of consciousness, no seizures, and no vomiting in due course. No foreign body was reported in the surrounding environment. Physical examination did not identify any abnormality. A cervical spine radiograph (Figure 1) was performed and showed a diffuse non-specific soft tissue thickening at the level of C1-C2 vertebrae.

In the setting of head trauma and according to the PECARN and ACR recommendations, the boy was classified as very low risk for clinically important brain injury, which makes head CT scan evaluation not appropriate [9,10]. He was then discharged from the hospital with a pain management treatment consisting of paracetamol and ibuprofen.

His condition worsened, and the boy was eventually admitted to the hospital five days later because of left-sided torticollis. Upon admission, he was febrile at 38.2°C. Nevertheless, according to the parents, his appetite and general condition were well-preserved. Clinical examination was unremarkable aside from some infra-centimetric cervical lymph nodes. A blood sample (Table 1) documented mild anemia (Hb = 10.4 g/dL) and an inflammatory response (C-reactive protein [CRP] = 52 mg/L and WBC = 15.840 K/uL). Even though rapid strep test and cultures for Streptococcus pyogenes, aerobic, and anaerobic bacteria were all negative, antibiotics (amoxicillin/clavulanic acid) were instituted, fearful of a retropharyngeal abscess. The child was hospitalized for IV antibiotic treatment.

**Table 1:** Laboratory results upon admission. Hb: Hemoglobin; Hct: Hematocrit; CRP: C-reactive protein; MPV: Mean platelet volume.

Indicator	Unit	Patient’s results	Reference range
Hb	g/dL	10.4	11.0-13.7
Hct	%	31.4	38.0-48.0
RBC	x10^6^/mm^3^	4.0	4.7-6.1
CRP	mg/L	52	<1
WBC	x10^3^/mm^3^	15.84	5.000-10.000
Neutrophils	x10^3^/mm^3^	10.12	2.500-6.000
Lymphocytes	x10^3^/mm^3^	4.01	1.000-4.000
Monocytes	x10^3^/mm^3^	1.41	0.2-0.8
Eosinophils	x10^3^/mm^3^	0.23	0.05-0.3
Basophils	x10^3^/mm^3^	0.07	0-0.1
Platelets	x10^3^/mm^3^	442	200-500
MPV	fL	8.3	7-12

**Figure 2:**
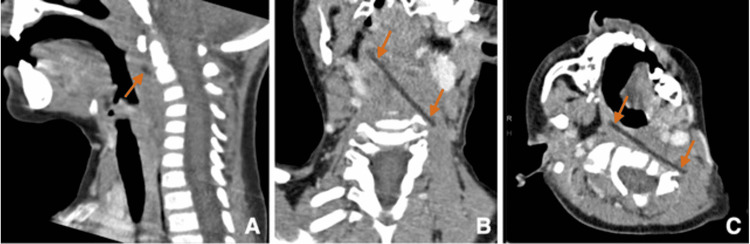
Sagittal (A), coronal oblique (B), and axial oblique (C) reconstructions of contrast-enhanced CT scan illustrating a 5.8-cm long, linear hypodensity located in the prevertebral soft tissues, along with an inversion of the cervical spine lordosis.

A contrast-enhanced CT scan of the head and neck (Figure 2) was performed. It revealed a 5.8-cm long, linear hypodensity extending cranially from the right prevertebral space at the level of C2, passing through the midline to abut left at the level of the C5-C6 space, suggesting a foreign body. Besides, the reversal of the physiological lordosis of the cervical spine was noteworthy.

**Figure 3:**
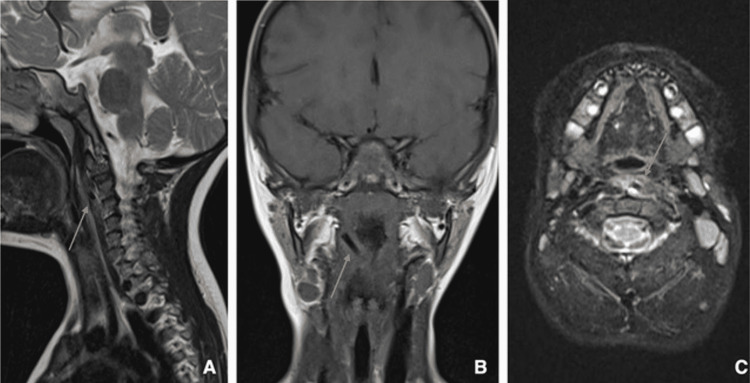
Sagittal T2-weighted (A), coronal T1-weighted (B) and axial T2-weighted with fat saturation (C) planes of the head and neck MRI. (A) Cranial portion of the foreign body (orange arrow) perforating the left longus colli muscle associated with high intensity T2-weighted changes within the muscle; (B) Right extremity of the foreign body (orange arrow); (C) Infiltration and thickening of the left longus colli muscle near to the foreign body (orange arrow).

The day after, a head and neck MRI with contrast medium (Figure 3) under sedation was performed, intending to evaluate the state of neighboring soft tissues and rule out any consequent damage. The foreign body was clearly visualized, penetrating the left longus colli muscle. Prevertebral soft tissue thickening and inflammatory changes were distinguished, with moderate narrowing of adjacent central airways. Internal and external carotid arteries were intact on both sides and the vertebral arteries. In fact, the left vertebral artery dodged the foreign body by 2 millimeters. The spinal cord was equally spared.

**Figure 4:**
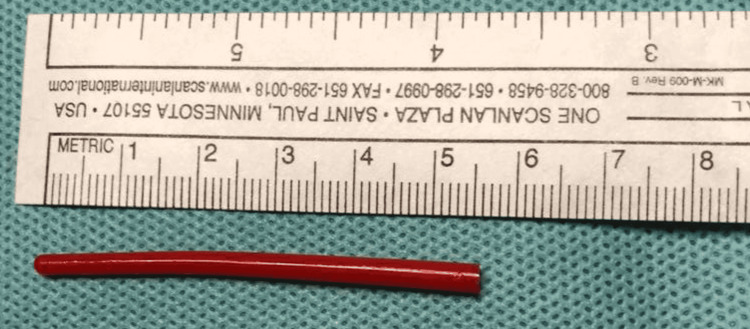
Photograph of the plastic paintbrush fragment upon retrieval, measuring 5.5 cm.

Finally, surgical extraction of the foreign body was carried out under general anesthesia without any immediate postoperative complication. The foreign body turned out to be a plastic paintbrush fragment (Figure 4), measuring 5.5 cm. The child continued the antibiotic course, and postoperative follow-up showed complete regression of cervical stiffness and inflammatory syndrome. However, the patient recovered without any sequelae.

## Discussion

The presented case illustrates an unusual foreign body impaction on several levels. First, the three-year-old patient is not able to verbalize his neck pain. Second, no history of foreign body in the surroundings was reported at the time of the trauma. Third, the head trauma was minor and did not meet the evidence-based medicine criteria for further imaging evaluation of the head or of the cervical spine [7,8].

Even though foreign body incorporation might seem obvious in certain circumstances (e.g., impalement injuries, gravel debris in superficial wounds), it can sometimes go unnoticed [1]. In both events, the management of foreign bodies relies on imaging techniques in which radiation exposure, cost, availability, and patient-specific limitations must be reviewed [1,2].

In an intent to choose the best imaging modality, it is of significant value to gather information about the patient's medical history and conduct a meticulous clinical examination [1]. The choice of imaging tools depends on the chemical composition of the suspected foreign body and its presumed location [1]. Foreign bodies frequently include metal, glass, plastic, wooden, and other vegetative materials such as thorns or ceramics [5].

Guidelines for assessing children with head trauma are well-established by the ACR based on the PECARN assessment and classification [7, 8]. According to the PECARN, children with a Glasgow Coma Scale (GCS) of 15 and no signs of basilar skull fracture (retro-auricular bruising, peri-orbital bruising, hemotympanum, cerebral spinal fluid otorrhea or cerebral spinal fluid rhinorrhea), no loss of consciousness, no vomiting, no severe injury mechanism, no severe headache, and no signs of altered mental status are classified at very low risk for traumatic brain injuries [7,8]. Therefore, the ACR considers that this category of children can forgo CT evaluation of the head for acute head trauma. This was the case of our three-year-old patient, who presented initially with a perfect GCS score and none of the six cited symptoms. Nonetheless, the ACR considers it appropriate to undergo a CT or MRI evaluation of the head in the event of cognitive or neurologic signs in the subacute phase [7]. No mention is made of infectious signs or the cervical level. 

Plain radiographs are routinely used to detect foreign bodies. The main drawback of a plain radiograph is its inability to reveal non-opaque foreign bodies [3]. In fact, most plastic foreign bodies are radiolucent, and this attribute makes them difficult to be disclosed on radiographs within the pediatric airway [3]. Similarly, the plastic foreign body in our case was radiolucent, although non-specific soft tissue changes were noted on the initial neck radiograph (Figure 1). Additional imaging techniques must be implemented to detect such foreign bodies. However, foreign plastic bodies may appear on X-rays if they contain radiopaque portions, paint, decoration, or coating. For example, Kargl S et al. reported a plastic pin that happened to be visualized in the right lung of a 17-year-old boy, on a coronal radiograp, by virtue of its radiopaque metallic segment [9]. Another case is described by Hunter TB and Taljanovic MS, involving a coin-like plastic wafer that was spotted on a lateral view radiograph in a one-year-old girl [3].

Ultrasonography (US) is the most sensitive modality when the foreign body is located within the superficial tissues [2]. Other assets of US include its increasing availability and its lower cost compared to CT and MRI [2]. Typically, plastic foreign bodies are hyperechoic and show posterior reverberation [2,3]. Therefore, US was not used in our case because of the lack of suspicion of a foreign body. Ultimately, a neck ultrasound is not of high yield in such cases since depth is a limiting factor for US studies [2]. Besides, a head ultrasound was not done because it is not considered to have an essential role in head trauma imaging [7].

CT scan is the gold standard technique for reproducing foreign bodies' shape, density, and size [2,9]. CT scan is equally essential for deep foreign body imaging [2]. By establishing their exact localization, CT also facilitates the surgical removal of foreign bodies [2]. Although, CT involves a much larger amount of ionizing radiation than other methods [2]. Plastic foreign bodies are generally characterized by an attenuation level of 10-20 Hounsfield Units (HU) on CT scan and thus appear hypodense [2]. The retained paintbrush fragment had an attenuation level of -23 HU. CT scan findings were key in the diagnostic procedure in our case. Nevertheless, some types of plastic have similar attenuation levels to soft tissues, making them undetectable via CT scan when implanted in muscular tissues, for example [1,6]. In such cases, MRI can be superior to CT scan for detecting foreign bodies. As an illustration, Kartiko M et al. recently reported a case in which a 10-cm plastic foreign body lodging in the deep soft tissues of the pelvis was overlooked on a CT scan and was not identified until an MRI was performed [6]. 

However, MRI cannot be used as the first diagnostic modality if foreign bodies are suspected to be ferromagnetic. Ferromagnetic objects are subject to rotation and translation forces in the magnetic field and can undergo radiofrequency-induced heating, resulting in adverse events [1,3]. Since ferromagnetic materials are invariably radiopaque, it is advisable to perform X-ray or CT imaging prior to the MRI [1]. In the case we present, the paintbrush fragment displayed low-signal intensities on T1- and T2-weighted images, which is the norm for plastic foreign bodies in MRI studies [2]. 

Though most foreign body impaction cases require a simple extraction, some of them may pose a serious challenge. Few may even lead to irreversible damage resulting in morbidity and death [2,3]. Fortunately, the paintbrush fragment did not damage vital structures such as arteries or nervous elements. For example, mortality rates following vertebral artery injuries range between 3 and 19%, including carotid artery injuries [10].

As plastic is quite omnipresent in households, physicians must remain cautious and have a high index of suspicion even when confronted with normal plain radiographs. In fact, maintaining a high index of suspicion of foreign bodies is essential to ensure proper management [1]. One should remember that plain radiographs cannot effortlessly rule out foreign bodies of all kinds. Moreover, some referring physicians do not realize that plastic is not sufficiently opaque to be visualized on radiographs [3]. Therefore, multiple imaging techniques and strong knowledge in radiology are often required to rule out foreign bodies in children and assess any potential damage inflicted upon adjacent tissues.

## Conclusions

Diagnosis of radiolucent foreign bodies is sometimes laborious, especially in the pediatric population, because gathering history about the traumatic incident and conducting a proper physical examination are particularly hard. Since plastic is quite common in households, physicians must remain cautious and consider plastic and other radiolucent foreign bodies, even in minor traumatic contexts that do not meet further imaging evaluation criteria per ACR and PECARN recommendations. Withal, unremitting head and neck symptoms in children following a traumatic event should prompt the possible diagnosis of a retained foreign body. On top of that, special attention must be given to parents who repeatedly present their child in the wake of a traumatic event, regardless of a normal initial evaluation.
